# Supplementary material for: Exploration of Social Exclusion among Older Chinese Immigrants in the USA
Source: Int J Environ Res Public Health. 2023 Jan 31;20(3):2539. doi: 10.3390/ijerph20032539 (PMC9915086; doi:10.3390/ijerph20032539)
Supplement: Supplementary file 1 [file ijerph-20-02539-s001.zip › ijerph-2142031-supplementary.pdf]

International Journal of
Environmental Research and Public Health

MDPI

Supplemental Material

Interview Guide

A. Individual Domain

1. How do you perceive the differences, if there is any, between aging in America and in your country of origin?
2. What resources do you have or need in order to fulfill your aging? (This is intended to generate responses that will give rise to topics that the interviewer can follow.
3. What expectations does the respondent have about himself/herself to have a decent life in relation to his/her circumstance and what s/he thinks his/her family, cultural and broader social community expects of him/her?

B. Community Domain

1. Please tell me about the people in your life who you consider as friends (can be neighbors or friends) (Interviewer: Make note of how many friends there are and who they are helpful to draw out a diagram of who the friends are at this point. The rest of the probing questions will try to get at the respondent's perceptions about closeness, trust, etc. with each of the friends and in identifying the close friends, and how the closeness and trust etc. relate with aging of the participant.)
2. Please tell me about the activities you are involved in the community. (Probe for organizations where activities may take place, How did you initially become involved in doing the activity in above and why did you get involved?)
3. How do you think the community perceives Chinese seniors?
4. As a Chinese senior living in America, how do you feel like you belong here? what helps you feel like you belong?

C. Neighborhood Domain

1. Tell me about the neighborhood you live in (describe the neighborhood from the respondent's perspective) (e.g., rich neighborhood, poor neighborhood, recent immigrants, etc.)
2. How safe do you feel in your neighborhood? Why? Provide an example or two?
3. What do you like or dislike about your neighborhood? Why? Provide an example?
4. How well do you know your neighbors? Please tell me about them. OR Please tell me more about the relationships you have with your neighbors.
5. In your opinion, is your neighborhood deprived of resources? If so, please tell me in what ways this is happening.
6. From your perspective, what do you think of the role of neighborhood and the things you have mentioned about how your neighborhood affects your health and aging?

D. Services & Resources

1. In terms of services, such as health and social services, please tell me about your experiences of using these services. Probe for: positive experiences and what made them so; probe for negative experiences and what made them so. Important to probe for who was involved and what aspects of the interaction(s) made the experience positive or negative. What does the respondent do to promote and maintain positive experiences? What does the respondent do to decrease or avoid negative experiences?
2. What are the barriers for you to meet these expectations? Where do you access your health services?

3. Is/are these services outside of your neighborhood? Where? Please tell me about your sense of trust toward the health and social service system.
4. Please tell me to what extent these services and your experience with the service system affect your aging and health.
5. From your perspective as a Chinese senior in America, please tell me about your perception about the goods/resources to which you have access to achieve healthy aging in the way you had mentioned at the beginning of the interview. OR Tell me about the times when you have had positive experiences in accessing resources in the following areas Access to your cultural community for social activities, health care, employment, and other needs
6. Access to services outside of your cultural community for social services, health care, employment and other needs
